# Computed tomography for evaluation of abdominal wall hernias—what is the value of the Valsalva maneuver?

**DOI:** 10.1007/s10029-024-03036-6

**Published:** 2024-06-14

**Authors:** S. Ghafoor, A. T. Hoppe, M. Lange, A. Tognella, M. Bueter, K. Lehmann, H. Alkadhi, D. Stocker

**Affiliations:** 1 Institute of Diagnostic and Interventional Radiology, University Hospital Zurich, University of Zurich, Zurich, Switzerland; 2 Department of Visceral and Transplantation Surgery, University Hospital of Zurich, Zurich, Switzerland

**Keywords:** Hernia, Inguinal, Multidetector computed tomography, Valsalva Maneuver, Diagnostic imaging

## Abstract

**Purpose:**

To investigate the differences in the visibility and size of abdominal wall hernias in computed tomography (CT) with and without Valsalva maneuver.

**Methods:**

This single-center retrospective study included consecutive patients who underwent abdominal CTs with Valsalva maneuver between January 2018 and January 2022. Inclusion criteria was availability of an additional non-Valsalva CT within 6 months. A combined reference standard including clinical and surgical findings was used. Two independent, blinded radiologists measured the hernia sac size and rated hernia visibility on CTs with and without Valsalva. Differences were tested with a Wilcoxon signed rank test and McNemar’s test.

**Results:**

The final population included 95 patients (16 women; mean age 46 ± 11.6 years) with 205 hernias. Median hernia sac size on Valsalva CT was 31 mm compared with 24 mm on non-Valsalva CT (*p* < 0.001). In 73 and 82% of cases, the hernias were better visible on CT with Valsalva as compared to that without. 14 and 17% of hernias were only visible on the Valsalva CT. Hernia visibility on non-Valsalva CT varied according to subtype, with only 0 and 3% of umbilical hernias not being visible compared with 43% of femoral hernias.

**Conclusions:**

Abdominal wall hernias are larger and better visible on Valsalva CT compared with non-Valsalva CT in a significant proportion of patients and some hernias are only visible on the Valsalva CT. Therefore, this method should be preferred for the evaluation of abdominal wall hernias.

**Supplementary Information:**

The online version contains supplementary material available at 10.1007/s10029-024-03036-6.

## Introduction

An abdominal wall hernia is defined as a protrusion of contents from the abdominal cavity through a normal or abnormal aperture or focal area of weakness in the abdominal wall. Abdominal wall hernias can be present at different sites, including the inguinal, femoral, and umbilical region as well as along the linea alba, and at sites of prior incisions. Although the true prevalence is difficult to determine, abdominal wall hernias are one of the most common problems seen in general surgery practice [1]. Hernias can be symptomatic with pain and complicated by incarceration or strangulation [2]. Surgical repair is the treatment of choice for symptomatic disease and abdominal hernia repair is one of the most common surgical procedures performed worldwide [3, 4].

Diastasis recti (DR) is an abnormality of the anterior abdominal wall that can result in a midline abdominal bulge. It is characterized by a separation of the rectus abdominis muscles with concomitant widening of the linea alba. An intact rectus fascia differentiates DR from an abdominal wall hernia. Diastasis recti is common particularly in postpartum women and can coexist with or predispose to development of abdominal wall hernias. Symptomatic DR can present with pain and discomfort in the abdomen and musculoskeletal problems like pelvic instability, lumbar back pain, and pelvic floor disorders [5, 6].

Clinical assessment remains the mainstay for diagnosing most of the abdominal wall hernias and DR [7, 8]. Nonetheless, imaging can assist in diagnosing clinically occult cases, for treatment planning prior to surgery by mapping the abdominal wall and in those instances where clinical examination may be difficult due to patient habitus or scarring [9, 10]. Furthermore, imaging can aid evaluating the extent of DR in relationship to fixed abdominal landmarks [9]. On imaging, measurement of the inter-rectus distance (IRD) is used to diagnose and quantify RD.

Computed tomography with Valsalva maneuver (Valsalva CT) was proposed as an imaging modality for the evaluation of abdominal wall hernias [11–15]. To date, only one study has investigated the added value of Valsalva CT for the evaluation of abdominal wall hernias [11]. However, the reference standard used in that study was not clearly described [11]. Furthermore, the influence of Valsalva CT on IRD measurements for the diagnosis of DR is not known. Although some authors suggest that IRD should be measured in the relaxed state rather than under the Valsalva maneuver as measurements could be altered considerably [9], this has not been formally studied to date. Hence, the added value of Valsalva maneuver during CT image acquisition for evaluation of abdominal hernias and DR needs to be determined.

Therefore, the primary purpose of this study was to investigate the differences in the visibility and size of abdominal wall hernias in computed tomography (CT) with and without Valsalva maneuver. The secondary purpose was to investigate the influence of Valsalva CT on measurements of the IRD. The primary endpoint was the difference of hernia size between Valsalva and non-Valsalva CT. The secondary endpoints were differences in hernia visibility, hernia contents, and width of the IRD between Valsalva and non-Valsalva CT.

## Materials and methods

The institutional review board and local ethics committee approved this single-center retrospective study (*BASEC-Nr: 2021-02464*). All patients provided written informed consent of general use of their anonymized data for retrospective research.

### Patient population

The departmental radiology information system was searched for all consecutive patients who had undergone a Valsalva CT for the evaluation of abdominal wall hernias and/or DR between January 2018 and January 2022. Inclusion criteria was availability of an additional CT of the abdomen and pelvis without Valsalva maneuver (non-Valsalva CT) within 6 months. Exclusion criteria were as follows: no or declined signed general informed consent for research-related use of anonymized patient data, no non-Valsalva CT within a 6-month interval, abdominal surgery between the two scans, and no adequate reference standard*.* A small subset of the patients (*n* = 21) was previously included in another study, where imaging diagnosis of groin hernias was investigated [14]. Demographic and clinical data (age, body mass index [BMI]) as well as data for the reference standard (clinical examination findings, intraoperative findings) were retrieved from the electronic medical records.

### Reference standard

Abdominal surgeons assessed all patients clinically. Either abdominal wall hernias and/or DR were diagnosed clinically by physical exam, or a suspicion was raised based on clinical findings. The Valsalva CT was performed to evaluate the extent of the suspected hernias and/or DR, to screen for the presence of additional abdominal wall hernia, and for treatment planning. We used a reference standard comprised of findings from clinical examination and/or intra-operative findings for the confirmation of hernias and DR. Abdominal surgeons from our hospital performed all surgical hernia repairs and abdominal wall reconstructions, the latter procedure sometimes in conjunction with plastic surgeons. Procedures included laparoscopic and open techniques.

### CT imaging

Patients were scanned on different state of the art CT scanners (SOMATOM Force, SOMATOM Definition Edge, NAEOTOM Alpha, Siemens Healthineers, Forchheim, Germany). Protocol details are outlined in Supplemental Table 1. All patients received a non-contrast CT of the abdomen and pelvis in supine position and images were acquired during a maximum Valsalva maneuver. Prior to the scan, patients were instructed and trained by the technologist on how to perform the maneuver. No oral or rectal contrast was given. The protocol parameters of non-Valsalva CT varied depending on the clinical question, however, all covered the entire abdomen and pelvis and were performed without Valsalva maneuver.

### Image analysis

Two radiologists (one senior resident, one board-certified radiologist) with 4 (reader 1, *A.T.H.*) and 5 (reader 2, *M.L.*) years of experience in radiology independently reviewed the Valsalva CT and non-Valsalva CT images side-by-side. The readers were blinded to the clinical information and surgical findings. For the quantitative analysis, the readers were asked to measure the size of the hernia sac and the hernia orifice on the Valsalva CT and non-Valsalva CT, respectively. For the qualitative analysis, the readers had to define the hernia contents (fat, bowel, bladder), score whether there were more hernia contents on the Valsalva CT than on the non-Valsalva CT, whether the hernia was better visible on the Valsalva CT, and whether the hernia was visible at all on the non-Valsalva CT.

A third radiologist (reader 3, *S.G.*, a board-certified radiologist subspecialized in abdominal radiology with 9 years of experience) measured the IRD at the level of the umbilicus and 3 cm above the umbilicus in each patient. The IRD corresponds to the linea alba and is used to confirm and quantify DR [16]. In addition to measuring the IRD, the reader also scored whether there was any bulging of the linea alba on Valsalva CT and non-Valsalva CT. A single reader was chosen for the IRD measurements as the goal was to investigate differences in measurements between Valsalva and non-Valsalva CT and not to evaluate for the detection rate of DR with CT or reproducibility of IRD measurements.

All images were reviewed on the hospital’s picture archiving and communication system (PACS).

### Statistical analysis

The statistical analysis was performed using SPSS (version 29.0, IBM Corp, Armonk, NY). Clinical data and readout results were evaluated using descriptive statistics. Continuous variables are presented as either mean ± standard deviation or median (interquartile range [IQR]) and categorical variables as numbers with percentages.

Normal distribution of data was tested with the Kolmogorov–Smirnov test. Differences in hernia size measurements and IRD measurements between Valsalva CT and non-Valsalva CT were tested with a Wilcoxon signed rank test. The inter-reader agreement for the measurements was tested with the intraclass correlation coefficient (ICC) based on a consistency type two-way mixed model and interpreted as follows: ICC < 0.5 = poor agreement, ICC 0.5–0.75 = moderate agreement, ICC 0.75–0.9 = good agreement, and ICC > 0.90 = excellent agreement [17]. Differences in the protrusion of the linea alba between Valsalva CT and non-Valsalva CT were tested with a McNemar’s test. Results from the qualitative analysis were analyzed with descriptive statistics. Two-tailed *p* values below 0.05 were considered to infer statistical significance.

## Results

### Patient population and reference standard

**Fig. 1:**
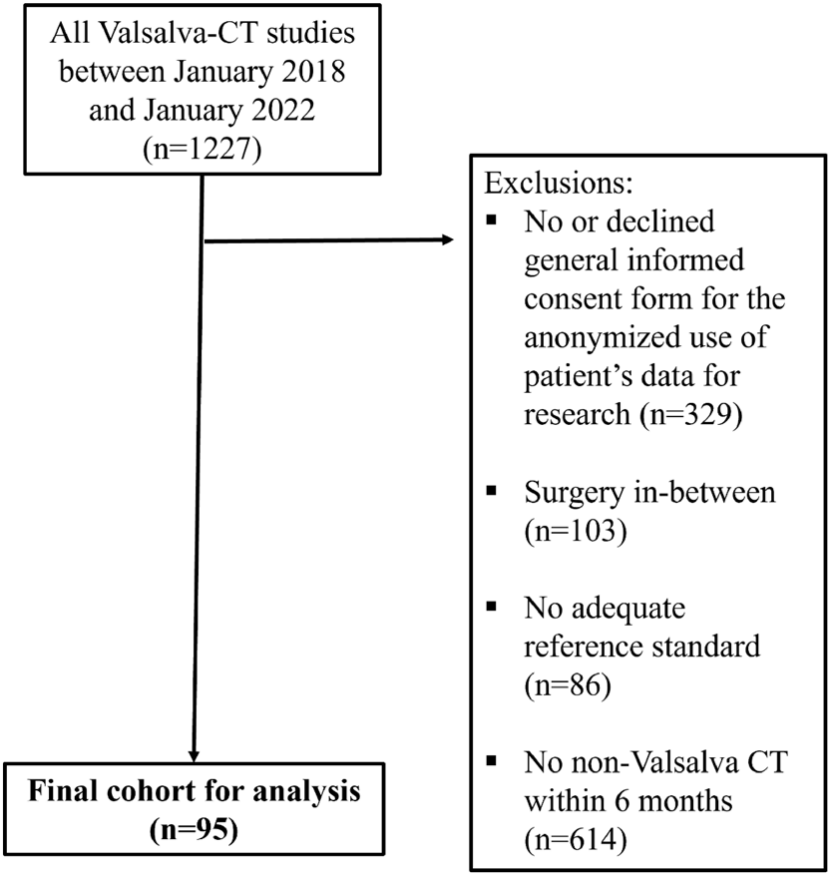
Flowchart of patient inclusion

The initial search in the RIS yielded 1227 eligible CT scans. After applying exclusion criteria, the final study population included 95 patients with 205 hernias (Fig. 1).

The median time between Valsalva CT and non-Valsalva CT was 57 days (IQR 33, 91). Main patient and hernia characteristics are outlined in Table 1.

**Table 1:** Patient characteristics

	Total population (*n* = 95)	Male patients (*n* = 79)	Female patients (*n* = 16)
Age (years)	46 ± 11.6	46 ± 11.9	61 ± 9.8
BMI (kg/m^2^)	26.0 ± 4.1	26 ± 4.4	25.6 ± 4.4
Number of hernias	205	168	37
Type of hernias			
Inguinal	97 (47%)	94 (5%)	3 (8%)
Femoral	40 (19%)	19 (11%)	21 (57%)
Umbilical	33 (16%)	26 (16%)	7 (19%)
Incisional	31 (15%)	27 (16%)	4 (11%)
Other^a^	4 (2%)	2 (1%)	2 (5%)
Surgically repaired hernias			
Inguinal	68 (70%)		
Femoral	34 (85%)		
Umbilical	17 (52%)		
Incisional	22 (71%)		
Other^a^	4 (100%)		
Clinical Diastasis recti^b^	10 (11%)	6 (7%)	4 (25%)

^a^Other includes parastomal and ventral hernias

^b^Patients with clinically evident and/or symptomatic diastasis recti

### Quantitative analysis: hernia size

The median hernia sac size measured by reader 1 was 31 mm (IQR; 21, 46) on Valsalva-CT and 24 mm (IQR; 16, 35) on non-Valsalva-CT (23% difference) (*p* < 0.001).

The median hernia sac size measured by reader 2 was 31 mm (IQR; 22, 45) on Valsalva-CT and 24 mm (IQR; 16, 35) on non-Valsalva CT (23% difference) (*p* < 0.001).

The median hernia orifice size measured by reader 1 was 19 mm (IQR; 12, 27) on Valsalva-CT and 14 mm (IQR; 9, 21) on non-Valsalva-CT (26% difference) (*p* < 0.001).

The median hernia orifice size measured by reader 2 was 21 mm (IQR; 14, 29) on Valsalva-CT and 16 mm (IQR; 11, 22) on non-Valsalva CT (24% difference) (*p* < 0.001).

Size measurements of hernia subgroups on Valsalva CT and non-Valsalva CT are depicted in Table 2 and relative size differences according to hernia subtype are depicted in Table 3.

**Table 2:** Hernia size measurements according to hernia subtypes

	Reader 1 (*n* = 205)				Reader 2 (*n* = 205)			
	Valsalva CT		Non-Valsalva CT		Valsalva CT		Non-Valsalva CT	
	Hernia sac	Hernia orifice	Hernia sac	Hernia orifice	Hernia sac	Hernia orifice	Hernia sac	Hernia orifice
Inguinal (*n* = 97)	33 mm (25, 45)	20 mm (14, 28)	22 mm (15, 33)	15 mm (9, 21)	34 mm (27, 46)	22 mm (16, 28)	24 mm (18, 34)	16 mm (11, 22)
Femoral (*n* = 40)	26 mm (21, 31)	14 mm (0, 25)	18 mm (16, 20)	10 mm (0, 13)	27 mm (23, 31)	20 mm (16, 25)	16 mm (0, 25)	14 mm (0, 18)
Umbilical (*n* = 33)	14 mm (12, 20)	8 mm (6, 14)	12 mm (9, 18)	7 mm (5, 10)	17 mm (12, 22)	10 mm (8, 17)	14 mm (10, 18)	8 mm (7, 11)
Incisional (*n* = 31)	43 mm (25, 70)	28 mm (16, 46)	36 mm (24, 57)	22 mm (10, 40)	46 mm (26, 27)	35 mm (19, 52)	33 mm (16, 42)	28 mm (13, 39)
Other^a^ (*n* = 4)	50 mm (31, 65)	22 mm (10, 36)	36 mm (29, 38)	15 mm (7, 26)	51 mm (32, 65)	34 mm (25, 40)	34 mm (25, 40)	16 mm (9, 26)

Data is presented as median and interquartile range in parentheses

^a^Other includes parastomal and ventral hernias

**Table 3:** Differences in hernia size and visibility in all hernias and according to hernia subtype

	Reader 1 (*n* = 205)		Reader 2 (*n* = 205)	
	Size difference^a^	Not visible on non-Valsalva CT	Size difference^a^	Not visible on non-Valsalva CT
All hernias (*n* = 205)	7 mm (23%)	34 (17%)	7 mm (23%)	28 (14%)
Inguinal (*n* = 97)	11 mm (33%)	13 (13%)	10 mm (29%)	9 (9%)
Femoral (*n* = 40)	8 mm (31%)	17 (43%)	11 mm (41%)	17 (43%)
Umbilical (*n* = 33)	2 mm (14%)	1 (3.0%)	3 mm (18%)	– (0%)
Incisional (*n* = 31)	7 mm (16%)	2 (6%)	13 mm (28%)	2 (6%)
Other^b^ (*n* = 4)	14 mm (28%)	1 (25%)	17 mm (33%)	– (0)

^a^Absolute differences with relative differences in parentheses of median maximum hernia sac size measured on Valsalva-CT and non-Valsalva-CT

^b^Other includes parastomal and ventral hernias

The ICC for measurements of the hernia sac were 0.98 (95% confidence interval [CI] 0.97–0.98) on Valsalva-CT and 0.95 (95% CI 0.93–0.96) for non-Valsalva-CT.

The ICC for measurements of the hernia orifice were 0.91 (95% confidence interval [CI] 0.88–0.94) on Valsalva-CT and 0.91 (95% CI 0.87–0.93) for non-Valsalva-CT.

### Quantitative analysis: hernia visibility and content

**Fig. 2:**
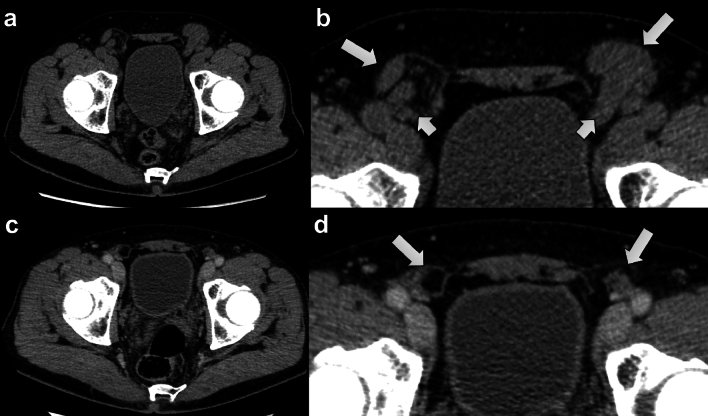
58-year-old male patient with bilateral inguinal hernias. Representative Valsalva CT image (**A**) and corresponding magnified image of the groin (**B**) shows bilateral inguinal hernias (long arrows in **B**) containing bowel loops (short arrows in B). On a non-Valsalva CT scan done 1 month later for pancreatitis (**C**, and magnified image of the groin in **D**) the hernias are barely visible (long arrows in **D**) containing only minimal fat

**Fig. 3:**
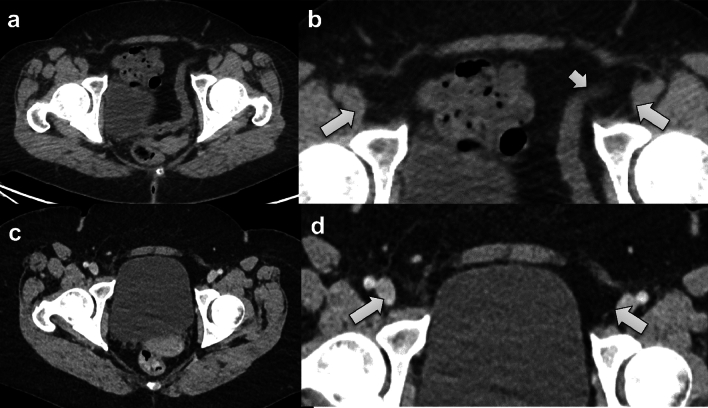
50-year-old female patient with prior Roux-en-Y gastric bypass and bilateral femoral hernias. Representative Valsalva CT image (**A**) and corresponding magnified image of the groin (**B**) shows bilateral femoral hernias (long arrows in **B**) with a protruding bowel loop on the left side (short arrow in **B**). A non-Valsalva CT scan (**C**, and magnified image of the groin in **D**) done for abdominal pain 3 months prior does not depict the right-sided femoral hernia clearly whereas the left-sided hernia is less conspicuous, and the protrusion of bowel is not depicted

Overall, the hernias were scored as better visible on Valsalva CT in 149 (73%, reader 1) and 169 (82%, reader 2) cases (Fig. 2). The contents of the hernia sac were larger on Valsalva CT in 59 (29%, reader 1) and 53 (26%, reader 2) cases (Fig. 3). Thirty-four (17%, reader 1) and 28 (14%, reader 2) hernias were not visible on non-Valsalva CT (Table 3).

Detailed results of hernia contents for reader 1 and reader 2 are outlined in Table 4. In 36 cases (54%, reader 1) and 37 cases (55%, reader 2) of hernias that contained bowel or bladder on Valsalva CT, only fat or no content at all (in those cases where the hernia was not visible) was seen on the corresponding non-Valsalva CT.

**Table 4:** Qualitative analysis of hernia contents on Valsalva-CT and non-Valsalva CT

	Reader 1 (* n* = 205)		Reader 2 (* n* = 205)	
	Valsalva CT	Non-Valsalva CT	Valsalva-CT	Non-Valsalva CT
None^a^	0	32 (16%)	0	28 (14%)
Fat	138 (67%)	141 (69%)	132 (64%)	141 (69%)
Bowel	64 (31%)	31 (15%)	66 (32%)	34 (17%)
Bladder	3 (2%)	1 (0.5%)	7 (3%)	2 (1.0%.)

^a^If the hernia was not visible, content was scored as “none” by readers

### Inter-rectus distance

**Fig. 4:**
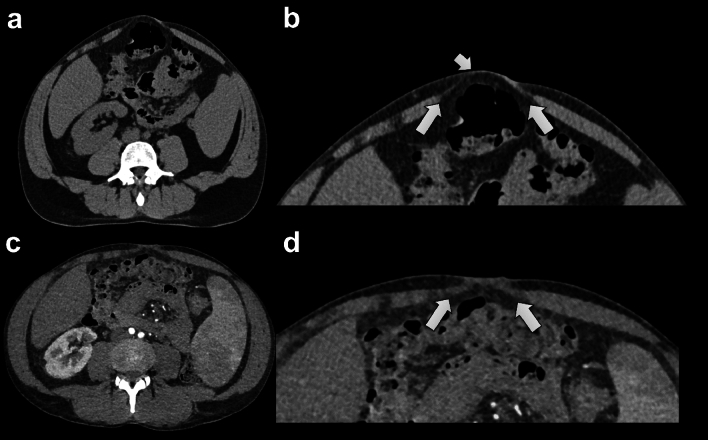
40-year-old male patient with symptomatic diastasis recti. Valsalva CT (**A**, **B**) showing widening of the linea alba above the umbilicus (long arrows in **B**) and bulging (short arrow in **B**). The inter-rectus distance was 64 mm. A non-Valsalva CT (**C**, **D**) done two months earlier for other reasons (to plan for endovascular aortic valve repair) does not depict the bulge and the inter-rectus distance appears smaller at 36 mm (long arrows in **D**)

In all patients, the median IRD at the level of the umbilicus was 23 mm (IQR; 16, 34) on Valsalva CT and 20 mm (IQR; 15, 33) on non-Valsalva CT (13% difference) (*p* < 0.001) (Fig. 4). The median IRD measured 3 cm above the level of the umbilicus was 22 mm (IQR; 15, 34) on Valsalva CT and 20 mm (IQR; 14, 31) on non-Valsalva CT (9% difference) (*p* = 0.004). The results of the IRD measurements in male and female patients are depicted in Supplementary Table 2.

In those patients with clinically confirmed or suspected DR (*n* = 10), the median IRD at the level of the umbilicus was 48 mm (IQR; 28, 58) on Valsalva CT and 39 mm (IQR, 25, 51) on non-Valsalva-CT (19% difference) (p=0.002). The measurements 3 cm above the umbilicus in this cohort were 52 mm (IQR; 30, 63) on Valsalva CT and 44 mm (IQR; 24, 60) on non-Valsalva CT (15% difference) (*p* < 0.001).

A bulging of the linea alba was seen in 17 patients (18%) on Valsalva CT compared with 10 patients (10%) on non-Valsalva CT (*p* < 0.001).

## Discussion

In this study, we evaluated the influence of the Valsalva maneuver on hernia visibility, hernia size and IRD compared with CT performed without Valsalva, using a combination of clinical examination findings and surgery as a reference standard for hernia confirmation. We found significant differences regarding hernia detection rate, hernia contents, hernia size measurements and IRD measurements with Valsalva CT being superior to non-Valsalva CT.

On Valsalva CT, all hernias regardless of subtype, appeared bigger than on non-Valsalva CT. Notably, the size difference was most pronounced for inguinal and femoral hernias (up to 41% difference) and least pronounced for umbilical hernias (up to 18% difference). These findings support the added value of Valsalva CT especially for the evaluation of groin hernias. On the other hand, our results suggest that there may be only limited added diagnostic value of an additional Valsalva CT in patients with umbilical hernias that were detected on a prior non-Valsalva scan.

More than half (54–55%) of those hernias that contained bowel or bladder, hence being at a potentially higher risk for complications, were either not visible or were only shown to contain fat on the corresponding non-Valsalva CT. These hernias were thus missed or rather “under-staged” on non-Valsalva compared to Valsalva CT.

Across all hernias, 14–17% were not visible on non-Valsalva compared to Valsalva CT. Again, there were variations according to hernia subtype, with 43% of femoral hernias not visible on non-Valsalva CT, whereas this was the case only for 3% of umbilical hernias. In the study by Jaffe at al. [11], 10% of hernias were not visible on the non-Valsalva scan, which is a slightly lower proportion than in our study.

There were significant differences in hernia size between Valsalva and non-Valsalva CT, and hernias were consistently measured larger on Valsalva CT. The inter-reader agreement for these measurements was excellent, indicating that Valsalva CT is superior to non-Valsalva CT for depiction of the maximal extent of hernias and thus likely providing a more accurate representation of the patient’s condition.

Our results also showed that hernias were better visible on Valsalva CT in most cases (73–82%). A study by Miller et al. [10] showed that a significant proportion of hernias were missed upon initial imaging (non-Valsalva scans) and were only correctly identified after a second dedicated review by a specialized radiologist. Their study underscores the importance of optimizing imaging protocols to maximize hernia detection. Our results support the use of Valsalva CT to maximize the diagnostic yield for abdominal wall hernias. Image interpretation and hernia detection could be facilitated with Valsalva CT given the better visibility and conspicuity of hernias.

To date, only one other study has investigated the role of the Valsalva maneuver on supine CT for the evaluation of abdominal wall hernias [11]. Overall, our findings are in agreement with the former study by Jaffe et al. [11], who examined CT with and without Valsalva maneuver in 100 patients for identifying abdominal wall hernias. Their study also found increased conspicuity and size of hernias with the Valsalva maneuver. However, there was no clear description of hernia subtypes and no subgroup analysis according to hernia subtype was reported. Notably, we found marked variations with regards to hernia conspicuity depending on hernia subtype. Furthermore, that study did not clearly describe the reference standard and the hernias had not been confirmed surgically, rather the studied imaging modality itself (the CT) was used as the reference, which is a limitation.

According to guidelines, the mainstay for hernia diagnosis remains clinical examination and ultimately, intraoperative exploration at the time of hernia repair [18]. In this setting, a routine preoperative CT scan is currently not recommended. However, the major benefit identified in this study seems a better preoperative identification of femoral hernia components which is particularly relevant in the workup of female inguinal pain. In this setting, our data indicate that Valsalva CT adds a diagnostic benefit in nearly half of the patients. Therefore, if imaging is considered for the evaluation of inguinal pain, it should be performed under Valsalva maneuver to maximize the detection rate for hernias.

For patients with an incisional hernia, the current guidelines recommend using CT or MRI for preoperative planning, especially cases with large or complex hernias [19]. According to expert consensus, preoperative CT enables better understanding of the anatomy of the hernia, assessment for possibility of primary fascial closure, visualization of the quality and degree of retraction of the rectus muscles, and provides optimal information for surgical planning [19–22]. In our study, the orifice and size of incisional hernias were measured bigger on Valsalva compared to non-Valsalva CT (up to 28% difference). It is important to obtain accurate information on the size of the hernia defect to evaluate for the possibility of tension-free primary fascial closure during incisional hernia repair which can have a significant impact on prognosis [19, 22, 23].

Our data shows that less hernias are missed whereas maximum hernia size and hernia contents are better depicted with Valsalva CT. Therefore, when cross-sectional imaging is obtained for the evaluation of abdominal wall hernia and/or evaluation of diastasis recti, it should be performed with a dedicated protocol under a Valsalva maneuver as this method is superior to non-Valsalva imaging. Particularly if hernia surgery is planned through a minimally invasive approach, identification of small defects separate from the main hernia can be relevant, e.g., for trocar placement or the choice of the mesh size.

Although our study shows that Valsalva CT increased hernia detection rate and provides more accurate information on hernia size and contents, it remains to be answered for which patients this modality could be most useful and how Valsalva CT impacts patient management and treatment decision making. Future research is needed to address these questions in order to define the most appropriate and beneficial use of Valsalva CT in different patient populations and clinical scenarios.

The definition, classification and management of DR are controversial in the literature [24, 25]. A range of symptoms is reported to be associated with DR, including body core instability, lumbar back pain, and urogynecological symptoms such as urinary incontinence, fecal incontinence, and pelvic organ prolapse [6]. A classification of DR based on the width of the defect (i.e., the IRD) has been proposed in the past [26]. However, there is not an established correlation of IRD measurements with patient’s symptoms [24, 27]. A cross-sectional CT study has shown presence of mild DR (based on IRD measurements) in 57% of an asymptomatic population undergoing CT for other reasons [28]. Nevertheless, IRD measurements are obtained to objectify the severity of DR to establish indication for surgery and for symptomatic patients with DR requiring surgical repair for reimbursement purposes as requested by some insurance companies, and our data suggest that Valsalva CT offers a better accuracy for determining the IRD [29]. Furthermore, diagnostic imaging can aid in those cases, where concurrent umbilical or ventral hernia is suspected, which can occur in up to 56% of men and 36% of women with DR [3, 30–32].

The IRD measurements were significantly higher on the Valsalva CT, although the median difference was only small (up to 3 mm) in the overall population. However, the median difference became greater in those patients with clinically evident DR (up to 9 mm, up to 19% difference). Furthermore, a bulge of the linea alba was more frequently evident on the Valsalva CT (18% compared to 10%). Some authors suggest that muscle contraction could obscure DR and patients should be examined in a relaxed state [9]. Our findings suggest that DR is better depicted on Valsalva CT than on non-Valsalva CT. However, it is crucial that patients are properly instructed on how to perform the maneuver correctly. Patients are asked to “push out” their belly as much as they can during the scan. Excessive muscle contraction and especially activation of the transversus abdominis muscle should be avoided, since it could obscure DR by reducing the IRD [9]. Nevertheless, our data indicate that optimal hernia evaluation and assessment of DR can be done with Valsalva CT alone without the need for additional scanning in the relaxed state. Notably, there is no standardized and objective method for assessing whether patients performed the Valsalva maneuver adequately based on the CT images.

Our study has some limitations. First, the Valsalva and non-Valsalva CT were not done at the same time, which is related to the retrospective nature of the study. However, a median time interval of 57 days between the two scans in our study suggests a very low likelihood of relevant interim changes. Second, our patient cohort is medium-sized, which is related to the retrospective nature and the use of stringent inclusion criteria. Third, this is a single-center retrospective study using data from a tertiary referral center and hence our findings may not be entirely representative of other clinical scenarios and patient populations. Finally, we did not correlate the measurements of the hernia size and IRD with intraoperative measurements, as these were not reported consistently. However, a prior study showed good correlation between CT and intra-operative measurements [33].

## Conclusion

Abdominal wall hernias are larger and better visible on Valsalva CT compared with non-Valsalva CT in a significant proportion of patients. These effects are especially pronounced for inguinal and femoral hernias. Furthermore, hernia contents are better seen with Valsalva CT. The method has an excellent inter-reader agreement. In addition, the inter-rectus distance is measured bigger and bulging of the linea alba is more frequently seen on Valsalva CT. Therefore, our study suggests that abdominal CT with Valsalva maneuver is superior to non-Valsalva CT for evaluation of patients with suspicion of abdominal wall hernias.

## Supplementary Information

Below is the link to the electronic supplementary material.

Supplementary file1 (DOCX 14 KB)

## Data Availability

All data supporting the findings of this study are available within the paper and its supplementary material.

## Abbreviations

95% CI: 95% confidence interval
BMI: Body mass index
CT: Computed tomography
DR: Diastasis recti
ICC: Intra-class correlation coefficient
IQR: Interquartile range
IRD: Inter-rectus distance
