# Supplementary material for: Computed tomography for evaluation of abdominal wall hernias—what is the value of the Valsalva maneuver?
Source: Hernia. 2024 Jun 14;28(5):1709–18. doi: 10.1007/s10029-024-03036-6 (PMC11449955; doi:10.1007/s10029-024-03036-6)
Supplement: Supplementary file 1 — Supplementary file1 (DOCX 14 KB) [file 10029_2024_3036_MOESM1_ESM.docx]

**Supplementary Table 1:** Technical details of the Valsalva-CT protocols.

| **Scanner** | SOMATOM Force, Siemens | SOMATOM Edge Plus, Siemens | NAEOTOM Alpha, Siemens |
| --- | --- | --- | --- |
| **Tube voltage (kVp)** | 150 | 110 | 120 |
| **Reference tube current-time product (mAs)*** | 80 | 120 | 102 |
| **Pitch factor** | 0.6 | 0.6 | 0.8 |
| **Slice thickness (mm)** | 2 | 2 | 2 |
| **Slice increment (mm)** | 1.5 | 1.5 | 1.5 |
| **Reconstruction kernel** | Br36d/ ADMIRE 3  (soft tissue) | Br38f/ ADMIRE 3  (soft tissue) | Br36/ QIR 3  (soft tissue) |
| **CT dose index (CTDIvol)**** | 6.94 ± 2.0 mGy | 8.69 ± 4.3 mGy | 5.1 ± 1.5 mGy |
| **Dose-length product (DLP)**** | 319.1 ± 101.5 mGy x cm | 224.2 ± 224.2 mGy x cm | 238.9 ± 89.2 mGy x cm |
| *Automated tube current modulation was used in each scan  ** Data is presented as mean ± standard deviation.  ADMIRE: Advanced modeled iterative reconstruction. QIR: Quantum iterative reconstruction | | | |

|  | **Female** (n=16) | | **Male** (n=79) | |
| --- | --- | --- | --- | --- |
|  | **Valsalva CT** | **non-Valsalva CT** | **Valsalva CT** | **non-Valsalva CT** |
| **Inter-rectus distance**  (at umbilicus) | 29 (20, 46) | 28 (20, 42) | 22 (16, 32) | 20 (15, 32) |
| **Inter-rectus distance** (3 cm above umbilicus) | 16 (14, 30) | 15 (13, 27) | 22 (16, 34) | 20 (15, 32) |
| Data is presented as median and interquartile range in parentheses.  * Others include parastomal and ventral hernias | | | | |

**Supplementary Table 2**: Measurements of inter-rectus distance in males and females
